# An Innovative Inexpensive Portable Pulmonary Edema Intubation Simulator

**DOI:** 10.21980/J8MM1R

**Published:** 2020-04-15

**Authors:** Joshua D Mastenbrook, Neil C Hughes, William D Fales, David T Overton

**Affiliations:** * Western Michigan University Homer Stryker M.D. School of Medicine, Department of Emergency Medicine, Kalamazoo, MI; ^ Western Michigan University Homer Stryker M.D. School of Medicine, Department of Internal Medicine, Kalamazoo, MI

## Abstract

**Audience:**

This pulmonary edema intubation simulator is designed to instruct paramedics, medical students, emergency medicine residents, emergency medical services fellows, and attending physicians.

**Introduction:**

Acute pulmonary edema results in respiratory distress and may require endotracheal intubation. On occasion, pulmonary edema can result in copious amounts of pink, frothy sputum in the airway, complicating intubation by hindering the intubator’s view. Although airway management skills are frequently taught in a simulation setting, the frothy sputum seen in acute pulmonary edema is not easily replicated. Several articles have been published in reference to simulation model development for difficult airway management due to emesis obscuring the view of the glottic opening.^1^,^2^ There is, however, a scarcity of literature describing pulmonary edema airway management simulator construction, with only one other model identified on our review of the literature, which utilized cadavers, baking soda, vinegar, and red food coloring.^3^

In our simulation center, we teach a variety of learners who may be called upon to care for patients in acute pulmonary edema in their clinical practice, including medical students, residents from various specialties, practicing physicians and pre-hospital personnel. We wished to familiarize these trainees with the challenges associated with intubating patients with significant frothy secretions within the hypopharynx by developing a dynamic, realistic, portable and inexpensive model to simulate the airway manifestations associated with acute pulmonary edema.

**Educational Objectives:**

By the end of the session, learners will be able to: 1. Discuss the pathophysiology of, and immediate stabilization management steps for, acute cardiogenic pulmonary edema. 2. List the indications, contraindications, and risks associated with intubating a patient with acute cardiogenic pulmonary edema. 3. Demonstrate effective communication and teamwork skills to manage the airway of a simulated patient in respiratory distress due to acute cardiogenic pulmonary edema. 4. Successfully and safely intubate a simulated patient with a difficult airway due to visual obstruction from frothy pulmonary edema secretions.

**Educational Methods:**

We adapted a previously owned commercial airway task trainer simulator using an aquarium pump, tubing, an air stone, and an endotracheal tube. Pulmonary edema solution was created with glycerin, dish soap, (distilled) water and simulated blood. The solution and air stone are placed in one of the simulator’s lungs. Subsequently, turning on the aquarium air pump generates simulated pulmonary edema within the lung itself, which froths up and out of the trachea and into the hypopharynx, mimicking the gross pathophysiological process.

Learners complete pre-reading assignments prior to attending a small group didactic-practical session. Following a brief case discussion, led by the instructor, about the management of a patient in respiratory distress due to acute pulmonary edema, learners transition to a hands-on experience intubating the pulmonary edema manikin with the use of direct and video laryngoscopy, aided by a large bore Yankauer for suction and a bougie. Depending on the training level of the learners, the instructor will use judgment and may elect to demonstrate intubating the manikin using video laryngoscopy before the learners attempt the procedure. The authors recommend that the instructor use video laryngoscopy for teaching purposes so that all learners can visualize the intubation techniques (Yankauer, bougie) in the context of copious pulmonary edema fluid obscuring the glottis and surrounding airway structures.

The practical portion is dedicated solely to intubation, with one learner assuming the role of the intubator and another assuming the role of a respiratory therapist, while the other leaners observe and/or provide real-time feedback. Learners rotate through these aforementioned roles. To maintain efficiency of the simulation session and maximize the number of intubation attempts each learner receives, the session is designed to begin with a case discussion about the management of a patient with acute pulmonary edema up through the timepoint of successful intubation, followed by a practical portion where the learners perform multiple intubations on the innovative pulmonary edema airway management task trainer. During the practical portion, real-time constructive feedback is given to each learner. At the end of the simulation session, a debriefing is completed.

This model can be used to address several ACGME Emergency Medicine Milestones,^4^ specifically Milestone 9 (General Approach to Procedures – PC9), Level 4 (Performs indicated procedures on any patients with challenging features [eg, poorly identifiable landmarks, at extremes of age or with comorbid conditions], and also Milestone 10 (Airway Management – PC10), Level 4 (Performs airway management in any circumstance taking steps to avoid potential complications). This model can also be used to address ACGME Emergency Medical Services Milestones,^5^ specifically “Procedures Performed in the Pre-hospital Environment – Patient Care,” Level 4 (Performs indicated procedures on any patients, including those with challenging features (eg, poorly identifiable landmarks, at extremes of age or with co-morbid conditions).

**Research Methods:**

At the conclusion of the session, verbal feedback is sought from each participant by the instructor: How helpful did you find this simulation experience for learning about airway management in patients with acute pulmonary edema? Did you find the pulmonary edema intubation model to be realistic? Following this simulation experience, how would you rate your personal confidence in terms of managing an airway complicated by acute pulmonary edema?

**Results:**

For under fifty dollars, we have been able to adapt one of our previously owned airway management task trainers to build a pulmonary edema intubation simulator. It has been used in a wide variety of settings for different learners, including medical students, residents, fellows and pre-hospital providers. Since the 2016–2017 academic year, two hundred and twenty-six emergency medicine residents (PGY1, PGY2, and PGY3) have successfully used our innovative pulmonary edema airway management task trainer. Qualitatively it has been well-received and felt to be realistic by both our learners and instructors based on verbal feedback received following the simulation sessions.

**Discussion:**

We are aware of only one prior report attempting to simulate the frothy sputum seen in acute pulmonary edema. Lipe, *et al*., described mixing baking soda, vinegar and red food coloring in a cadaver hypopharynx just prior to an intubation attempt.^3^ This combination creates a fizzy frothy solution that fills the hypopharynx and pushes proximally into the mouth. This model is limited by design, however, in that it was unable to mimic a true in vivo appearance of a continuous flow of pulmonary edema-like fluid from the glottic opening. We feel we were able to overcome this limitation and also believe it is important for the leaner to experience the challenges of intubation when faced with copious secretions originating from within the lower airways. Our model generates the froth from within the lung itself, and it migrates proximally, similar to the dynamic pathophysiological process that occurs in vivo. Since we did not compare these two techniques, it is unknown which is more realistic. Neither the Lipe cadaver model nor our manikin model has been validated in terms of the realistic nature of the simulated pulmonary edema fluid. This would be ripe for future investigation. Nonetheless, informal qualitative feedback from our learners and instructors has been positive.

Resident use of our innovative dynamic pulmonary edema airway management task trainer has been incorporated into our Emergency Medicine residency and Emergency Medical Services fellowship Clinical Competency Committee discussions with respect to ACGME Milestone satisfaction. Our model addresses level 4 of Emergency Medicine Milestone 9 (General Approach to Procedures) and Milestone 10 (Airway Management). Additionally, level 4 of Emergency Medical Services Milestone 2 (Procedures Performed in the Pre-hospital Environment – Patient Care) is addressed. Incorporating successful intubation of the dynamic pulmonary edema airway management task trainer has provided the EM and EMS faculty with a more objective measure by which to score the aforementioned milestones during the mid-year and year-end Clinical Competency Committee meetings.

Overall, this innovation has met our objectives well. We have added this model to our library of more complicated airway management scenarios, such as vomitus and aspiration. Our emergency medicine residency program hosts a version of the difficult airway course and includes this pulmonary edema simulation station as part of that course. The model is very portable, allowing us to transport it to different sites for use. It is inexpensive, costing less than $50 to construct. Finally, the design is readily adaptable to any standard airway training manikin that has a simulated hollow lung with a detachable connection to a conduit representing a bronchus, which has a direct connection with a simulated trachea into which an endotracheal tube can physically be passed.

**Topics:**

Airway management, difficult airway, intubation, obstructed airway, pulmonary edema, video laryngoscopy, visual obstruction

## USER GUIDE

[Table t1-jetem-5-2-i9]

**Table t1-jetem-5-2-i9:** 

List of Resources:	
Abstract	9
User Guide	13
Instructor Guide	19

**Learner Audience:**

Medical Students, Interns, Junior Residents, Senior Residents, Paramedics, Attending Physicians

**Time Required for Implementation:**

**Preparation:** Approximately 20–30 minutes: 10 minutes to create the bubble solution, 10–20 minutes to modify the airway management task trainer. The simulated pulmonary edema bubble solution must be prepared at least 24 hours ahead of the session to allow the mixture to settle.

**Didactic-Practical session:** Approximately 45–60 minutes: 15 minutes to discuss the pathophysiology and management of acute cardiogenic pulmonary edema, 30–45 minutes to practice intubation.

**Recommended Number of Learners per Instructor:**

3–4 learners per instructor

**Topics:**

Airway management, difficult airway, intubation, obstructed airway, pulmonary edema, video laryngoscopy, visual obstruction

**Objectives:**

By the end of the session, learners will be able to:

1. Discuss the pathophysiology and immediate stabilization management steps of acute cardiogenic pulmonary edema
2. List the indications, contraindications, and risks associated with intubating a patient with acute cardiogenic pulmonary edema
3. Demonstrate effective communication and teamwork skills to manage the airway of a simulated patient in respiratory distress due to acute cardiogenic pulmonary edema
4. Successfully and safely intubate a simulated patient with a difficult airway due to visual obstruction from frothy pulmonary edema secretions

### Linked objectives and methods

In order to provide our learners with the opportunity to develop knowledge and skills for intubating a difficult airway with a dynamic flow of copious pulmonary secretions in a controlled low-stakes environment, we modified a currently owned airway management task trainer. Learners are expected to complete pre-reading assignments and actively participate in discussion about the pathophysiology and management of acute cardiogenic pulmonary edema (objective 1). The discussion concludes with the topic of intubation of patients in respiratory distress secondary to acute cardiogenic pulmonary edema, including the indications, contraindications, risks, and procedural techniques (objective 2). During the practical portion of the simulation session, learners work in pairs – physician intubator and respiratory therapist – to manage the difficult airway while the other 1–2 learners and the instructor provide constructive feedback (objective 3). Time is allotted for each learner to complete 3–5 intubations (objective 4).

### Recommended pre-reading for instructor

1. DuCanto J, Serrano KD, Thompson RJ. Novel airway training tool that simulates vomiting: Suction-Assisted Laryngoscopy Assisted Decontamination (SALAD) system. *West J Emerg Med*. 2017;18(1):117–120. doi:10.5811/westjem.2016.9.30891.
2. Weingart SD and Bhagwan SD. A novel set-up to allow suctioning during direct endotracheal and fiberoptic intubation. *J Clin Anesth*. 2011;23(6):518–519. doi:10.1016/j.jclinane.2010.08.021.
3. Sampson C, Pauly J, Horner J. Low-cost Portable Suction-Assisted Laryngoscopy Airway Decontamination (SALAD) Simulator for Dynamic Emesis. *J Educ Teach Emerg Med*. 2019;4(2):1–7.
4. Weingart S and DuCanto J. “Having a vomit SALAD with Dr. Jim DuCanto-Airway management technique during massive regurgitation, emesis or bleeding.” *EM Crit-*RACC Podcast196. 3 Apr. 2017. EMCrit, https://emcrit.org/emcrit/having-a-vomit-salad-withducanto/. Published April 3, 2017. Accessed December 31, 2019.
5. Bowman J. “SALAD Demonstration with the SSCOR DuCanto Catheter.” Video. https://www.youtube.com/watch?v=34jNKlH0ri4. Published November 2016. Accessed December 31, 2019.

### Learner responsible content (LRC)

1. Besecker B and Crouser E. Pulmonary edema. In: Vincent J, Abraham E, Moore F, Kochanek P, Fink M, eds. *Textbook of Critical Care*.7^th^ ed. Philadelphia, PA: Elsevier Inc.; 2017:38–41.
2. Nickson C. Suction assisted laryngoscopy airway decontamination (SALAD). *Life in The Fast Lane*. https://lifeinthefastlane.com/ccc/suctionassistedlaryngoscopy-airway-decontamination-salad/. Published August 21, 2018. Accessed December 31, 2019.
3. Patel K, Mastenbrook J, Pfeifer A, Bauler L. Successful Intubation of a Difficult Airway Using a Yankauer Suction Catheter. *J Emerg Med*. 2019;57(3):383–386. doi:10.1016/j.jemermed.2019.05.021.
4. Bowman J. “SALAD Demonstration with the SSCOR DuCanto Catheter.” Video. https://www.youtube.com/watch?v=34jNKlH0ri4. Published November 2016. Accessed December 31, 2019.

### Implementation Methods

Prior to the start of the simulation session, the instructor or a staff member will prepare the pulmonary edema manikin up to the point of turning on the aquarium air pump. One instructor and 3–4 learners begin the simulation session with a 15-minute discussion about the pathophysiology and management of acute cardiogenic pulmonary edema. The discussion wraps up with the instructor demonstrating (using a GlideScope Cobalt AVL) and sharing advice for intubating patients with visually obscured glottic openings. The extent of the demonstration is a judgment of the instructor based on the training level of the learners. During this latter portion of the discussion, the aquarium air pump is turned on to begin the process of simulated bronchorrhea. The learners then spend the next 30–45 minutes practicing intubation techniques on the simulated pulmonary edema manikin, with the goal of each learner completing 3–5 intubations. Both direct and video (GlideScope Cobalt AVL) laryngoscopy equipment are available and learners are encouraged to use both devices to become familiar with the advantages and disadvantages of each when managing an airway complicated by acute pulmonary edema. Additionally, learners become familiar with two intubation adjuncts, the large bore suction Yankauer, and the elastic gum bougie. While one learner assumes the role of physician intubator, another learner will assume the role of respiratory therapist, and the other 1–2 learners observe and provide feedback. Throughout the practical portion of the session, the instructor provides constructive feedback and at the end of the simulation session leads a debriefing and collects verbal feedback from the learners.

Clean-up of the simulation is relatively easy. The lung is disconnected from the manikin and the remaining fluid is discarded. The lung, air stone, and manikin airway structures (bronchus, trachea) are all rinsed under running warm water until the simulated pulmonary edema solution appears to have been flushed out of these components. Because the manikin bronchus and trachea remain attached to the manikin during this clean-up phase, it is easier to flush the airway if a smaller diameter tube is connected to the sink faucet and passed through the mouth and into the glottic opening. If this is not an available option, holding the distal end of the bronchus up to the sink faucet and allowing the warm water to travel cephalad and out the mouth will work well too. Angling the manikin to at least 45-degrees with the head positioned down in the sink will allow the water to easily run out of the manikin’s mouth and not travel back down the esophagus into the manikin’s stomach. To prevent mold from growing, the lung and manikin airway components are rinsed with a 10% bleach solution. The simulated blood is much less likely to stain than red food coloring. If any residual red hue is left on the outside of the manikin or tabletop, rubbing alcohol can be used to remove this. All components are then left to air dry.

### List of items required to replicate this innovation

To construct our pulmonary edema intubation simulator, we adapted a previously owned affordable, commonly available commercial airway task trainer (Laerdal® Airway Management Trainer, Wappinger Falls, NY) and purchased the additional following equipment:

1. Aquarium air pump (Tetra 77851, pet supply store). $7.49[Fig f1-jetem-5-2-i9]
  **Figure f1-jetem-5-2-i9:**
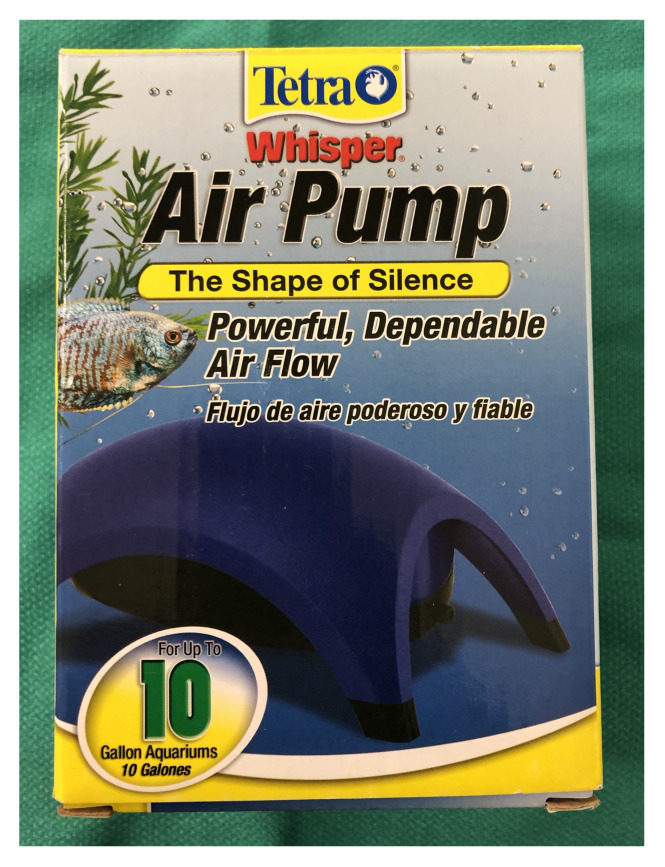
2. Air pump tubing (8 feet, pet supply store). $1.33[Fig f2-jetem-5-2-i9]
  **Figure f2-jetem-5-2-i9:**
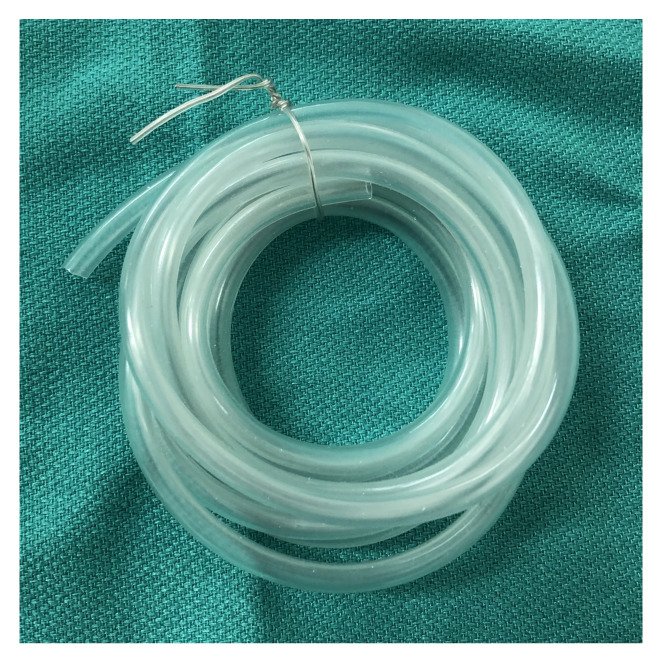
3. Aquarium air stone (pet supply store). $1.50[Fig f3-jetem-5-2-i9]
  **Figure f3-jetem-5-2-i9:**
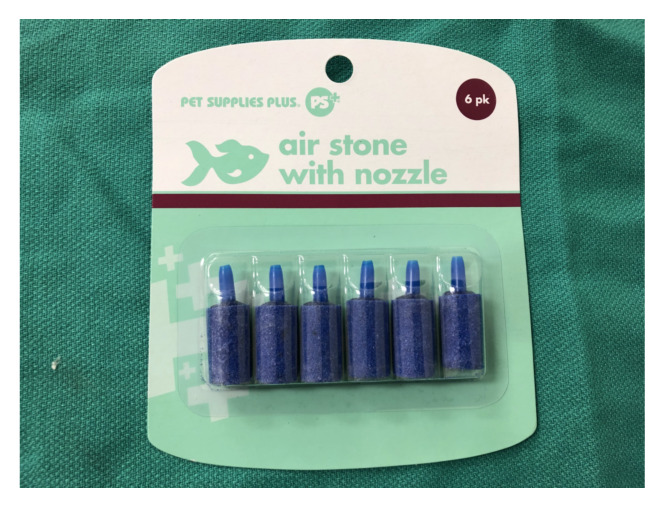
4. Endotracheal tube (size 3.0, uncuffed, shopmedvet.com). $2.29[Fig f4-jetem-5-2-i9]
  **Figure f4-jetem-5-2-i9:**
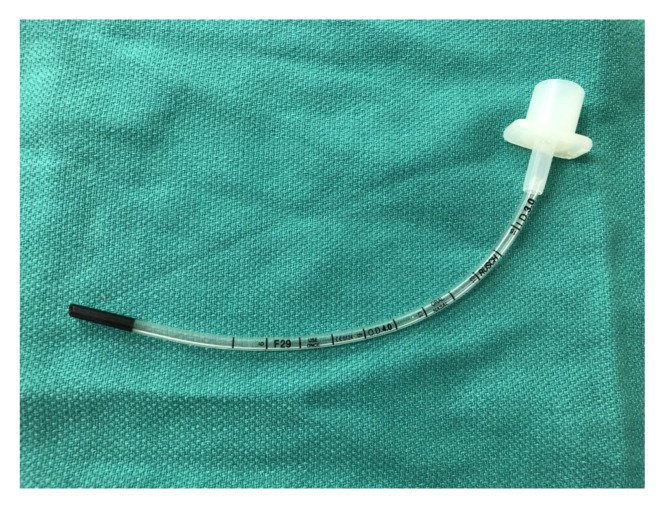
5. Glycerin (Beauty 360, 6 oz, CVS pharmacy). $7.29[Fig f5-jetem-5-2-i9]
  **Figure f5-jetem-5-2-i9:**
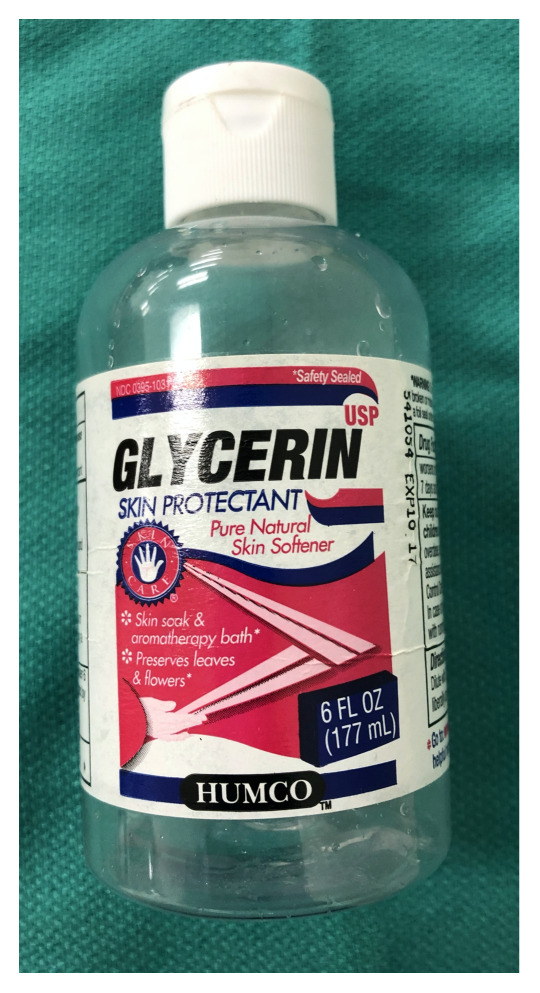
6. Simulated blood (3 oz, Laerdal®). Note that common red food coloring might permanently stain some manikins. $20.80[Fig f6-jetem-5-2-i9]
  **Figure f6-jetem-5-2-i9:**
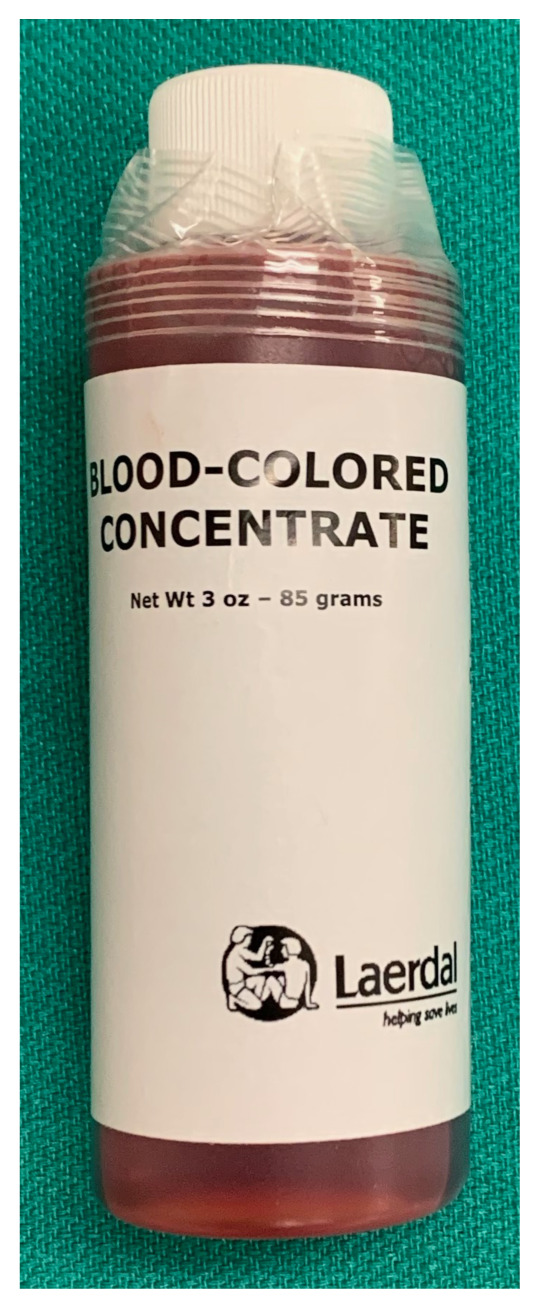
7. Dawn dish soap (blue, 56 oz, grocery store). $6.98[Fig f7-jetem-5-2-i9]
  **Figure f7-jetem-5-2-i9:**
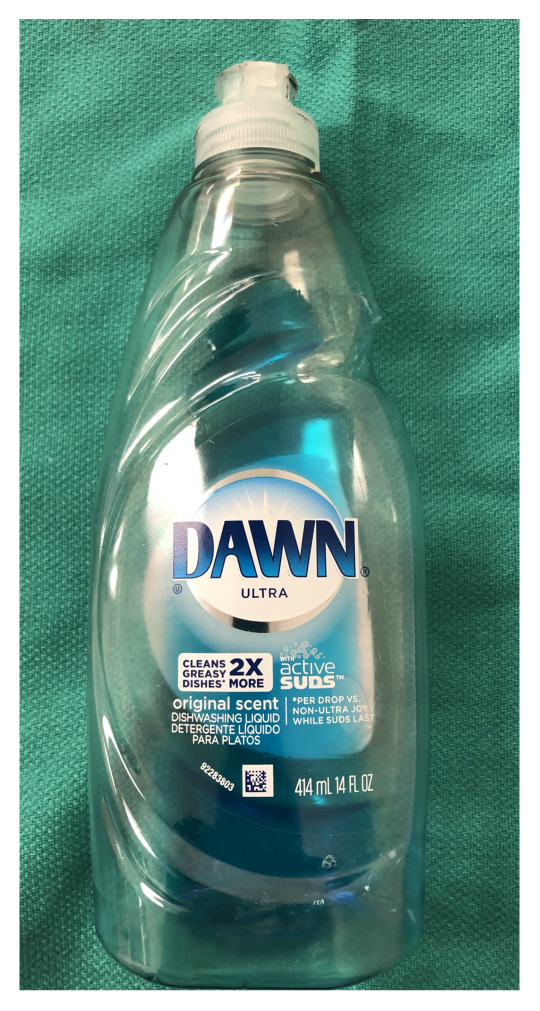

### Approximate cost of items to create this innovation

Cost of a new Laerdal® Airway Management Trainer: $2,215. Instructors may also be able to find used airway manikins at a discounted price at online retailers such as amazon.com, or repurpose aging manikins. The authors used a currently owned airway management task trainer, thereby necessitating only the purchase of the equipment to modify the manikin. Cost of equipment to modify our airway management task trainer to create a pulmonary edema simulator: $47.68.

### Detailed methods to construct this innovation

Bubble Solution:

1. Gently combine 250 cc (distilled) water, 30 cc Dawn dish soap, and 15 cc glycerin in a 500 cc glass beaker, or similar container. Stir cautiously to create a homogenous solution while minimizing the creation of soap bubbles.
2. Allow solution to sit undisturbed for at least 24 hours
3. Just prior to use, pour 30–50 cc of the bubble solution from the beaker into a squeeze bottle and add 2 small drops of the (Laerdal®) simulated blood. Gently rock or rotate the squeeze bottle to mix the bubble solution and simulated blood. The solution should now have a faint pink hue reminiscent of the color of true pulmonary edema. The 30–50 cc of solution will be added to one of the manikin’s lungs in a future step as noted below.

Modification of airway task trainer:

1. Disconnect one lung from the airway task trainer[Fig f8-jetem-5-2-i9]
  **Figure f8-jetem-5-2-i9:**
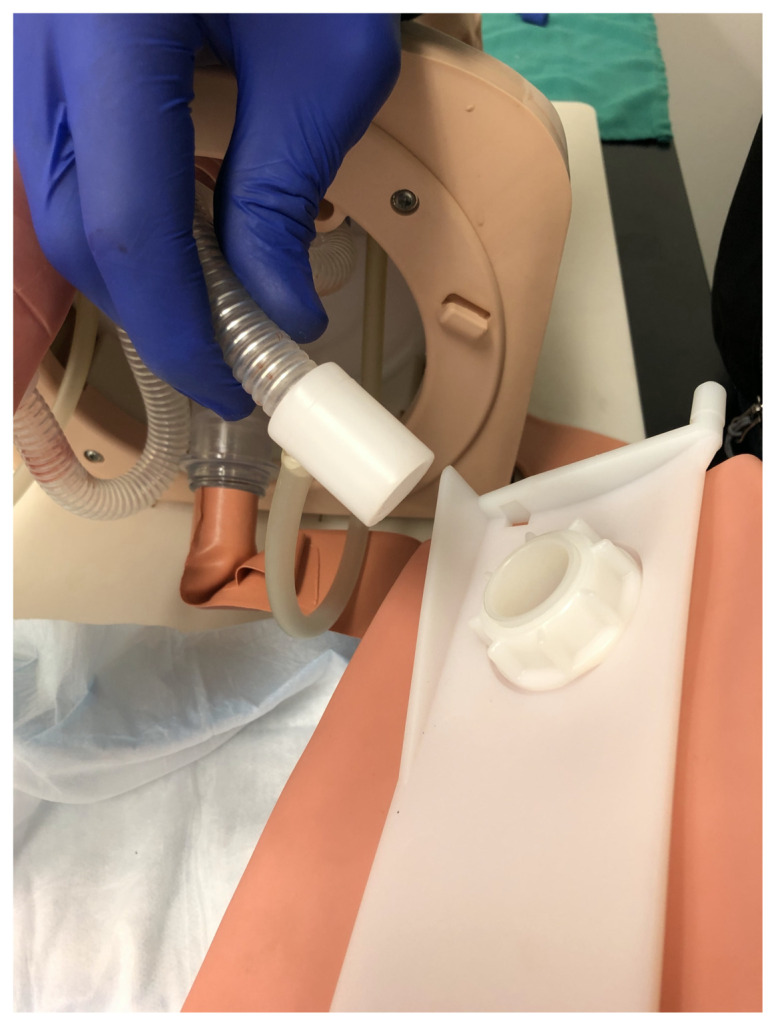
2. Add 30–50 cc of bubble solution into the airway manikin lung
3. Remove the plastic white lung connection adapter from the manikin bronchus[Fig f9-jetem-5-2-i9]
  **Figure f9-jetem-5-2-i9:**
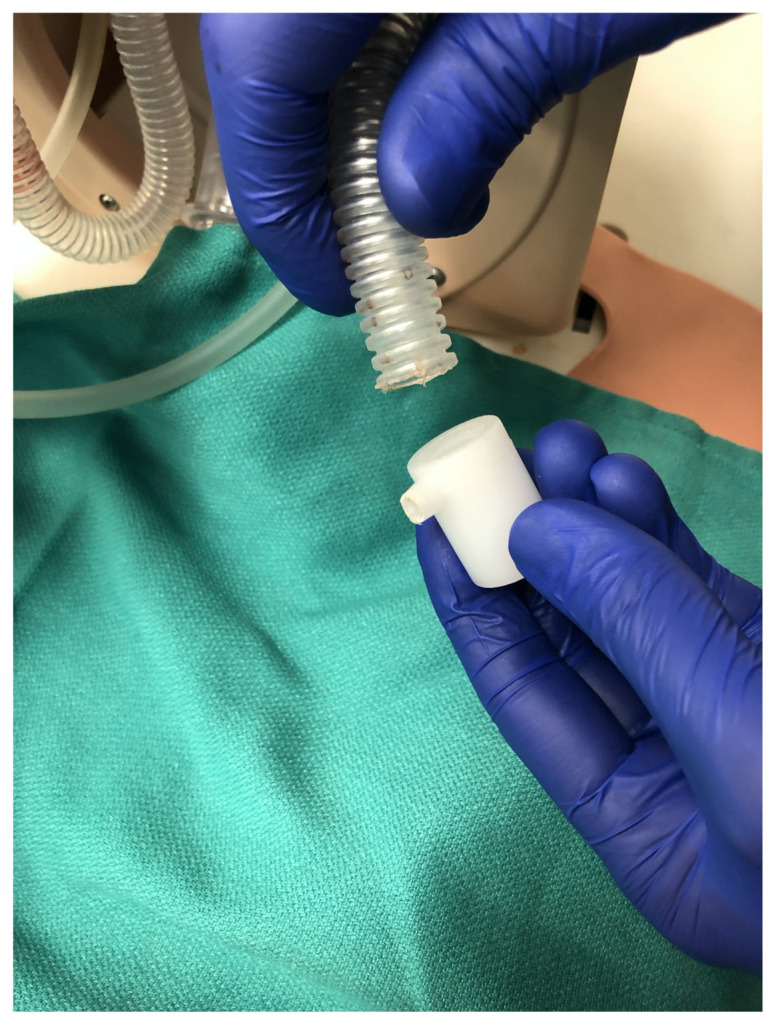
4. Remove the bag-valve-mask adapter from the proximal end of the endotracheal tube and feed the proximal end of the uncuffed 3.0 endotracheal tube through the side port of the manikin lung connection adapter in a caudal direction until there is approximately equal lengths of the endotracheal tube extending out from the side port of the adapter and from the caudal end of the adapter. Next, attach the air stone to the proximal end of the endotracheal tube via a 5–10 cm length of air pump tubing. Then, attach a 10–20 cm length of air pump tubing to the distal end of the endotracheal tube. Reconnect the manikin bronchus and white plastic lung connection adapter.[Fig f10-jetem-5-2-i9]
  **Figure f10-jetem-5-2-i9:**
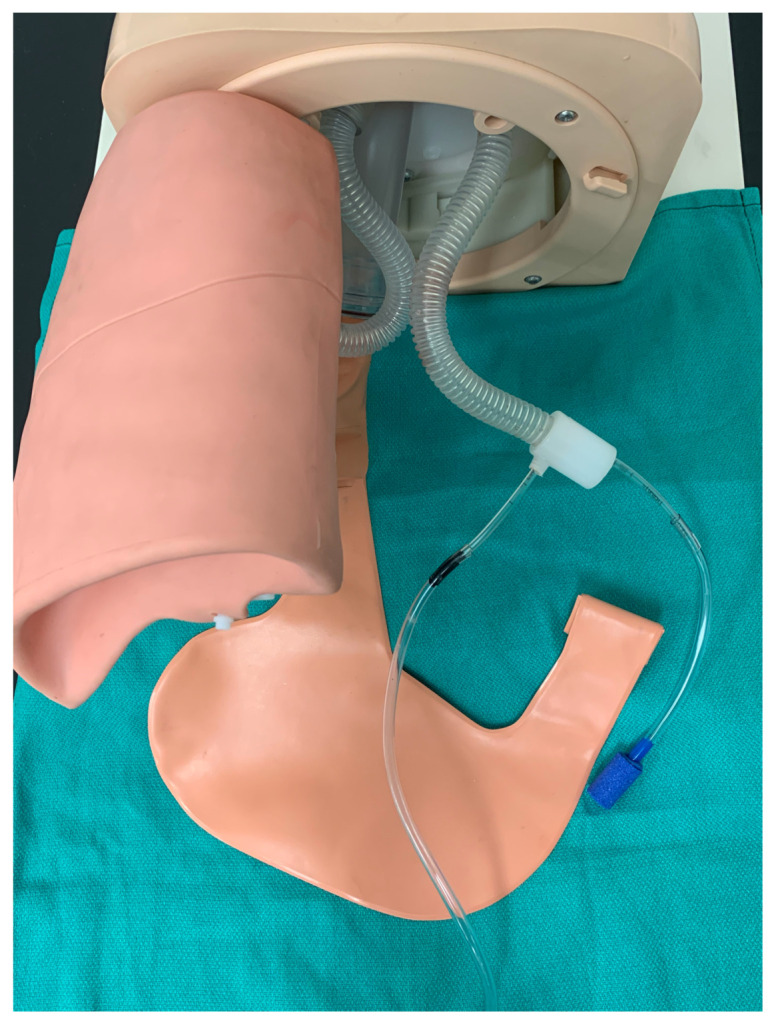
5. Connect the free end of the 10–20 cm length of air pump tubing to the aquarium air pump.
6. Feed the air stone into the manikin lung and then reattach the manikin lung to the airway task trainer.[Fig f11-jetem-5-2-i9]
  **Figure f11-jetem-5-2-i9:**
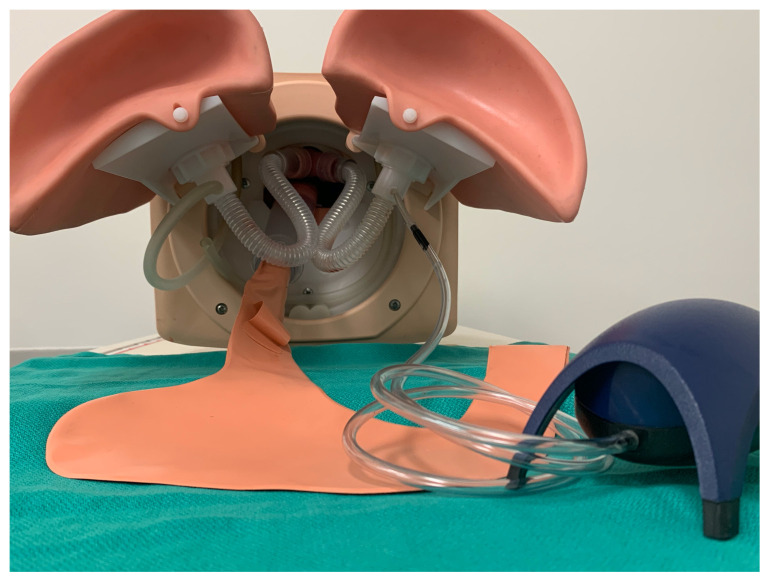
7. Turn on the aquarium air pump and allow several minutes for the pink frothy fluid to spread up into the hypopharynx in a continuous flow. Manually squeezing the lung can help start and speed up the initial spread of the fluid.[Fig f12-jetem-5-2-i9]
  **Figure f12-jetem-5-2-i9:**
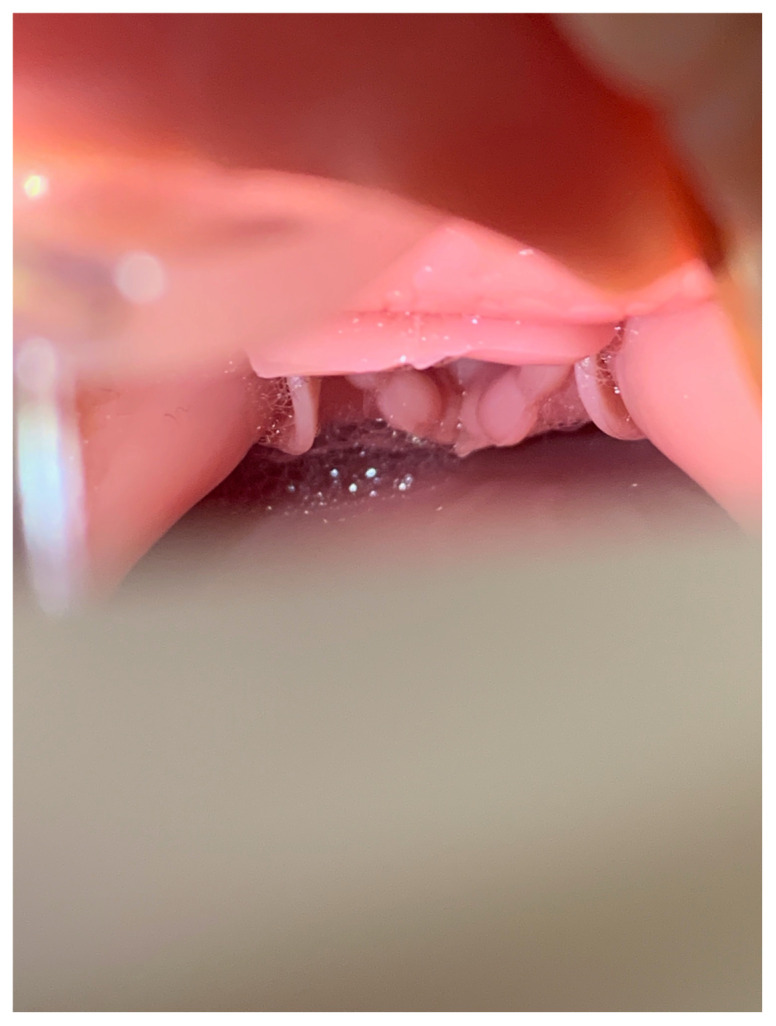
[Fig f13-jetem-5-2-i9]
  **Figure f13-jetem-5-2-i9:**
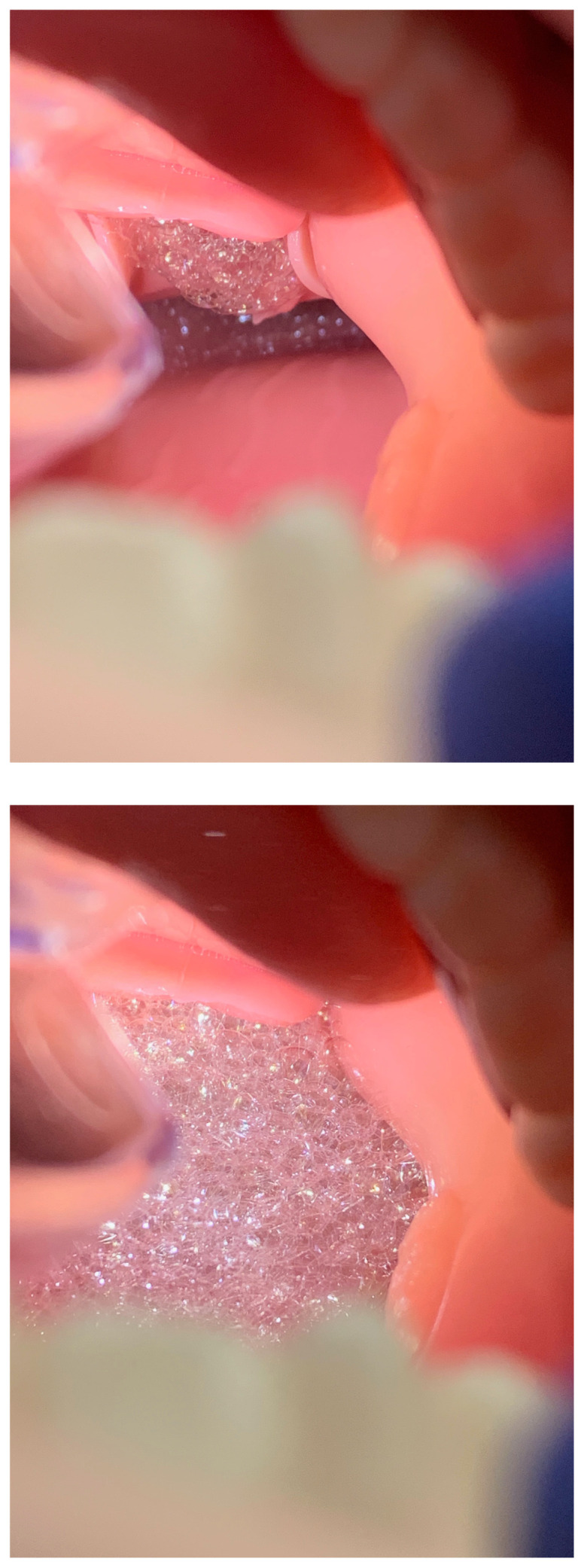

Video demonstrating the construction of the pulmonary edema simulator:

- Mastenbrook J and Hughes N. “Building a Pulmonary Edema Simulation Model.” Video. https://youtu.be/_zb1RvQNRoA. Published February 2018. Accessed December 31, 2019.
- https://youtu.be/2fNNlt463zs

### Associated content

Pulmonary Edema Manikin Simulation Didactic Instructor Guide: Key Points

### Results and tips for successful implementation

For under fifty dollars, we have been able to adapt one of our previously owned airway management task trainers to build a pulmonary edema intubation simulator. Additionally, with this highly portable setup, we were able to simulate acute pulmonary edema in a variety of clinical settings, including intubating on the floor, in tight spaces, on a stretcher, and in poor lighting conditions.

Based on feedback from learners and instructors, there were two modifications that we made to the simulation after its initial introduction. First, we added a few drops of red color to the bubble solution to create a more realistic appearance to the simulated pulmonary edema froth. Of note, red food coloring dye was difficult to remove from the manikin and tabletop on which the simulation took place. Switching the color additive to simulated blood resulted in no staining and easier clean-up. The manikin airway components and lung were simply run under warm water and allowed to dry. Second, it was noted to take more than a few minutes for the pulmonary edema froth to travel from the lung to the hypopharynx after initially starting the aquarium air pump. We found that manually squeezing the manikin lung after 1–2 minutes of starting the aquarium air pump sped up the flow of fluid into the hypopharynx. After this point, with just the aquarium pump left running, a continuous flow of froth out of the glottis was maintained in an adequate amount for the purpose of the simulation.

We have added this model to our library of more complicated airway management scenarios, such as vomitus and aspiration. At our institution, the emergency medicine residents participate in 10–12 simulation days throughout the academic year and will rotate through an intubation station 1–2 times each year. Additionally, our emergency medicine residency program hosts a version of the difficult airway course and includes this pulmonary edema simulation station as part of that course. Since the 2016–2017 academic year, two hundred and twenty-six residents have successfully used our innovative pulmonary edema airway management task trainer. The simulator has been well-received and reported to be realistic by our learners and instructors based on verbal feedback received following the simulation sessions.

## INSTRUCTOR GUIDE

### Pulmonary Edema Manikin Simulation Didactic Instructor Guide: Key Points

**Duration**: 15 minutes

**Maximum student to instructor ratio**: 4:1

**Objectives**:

1. Discuss the pathophysiology and immediate stabilization management steps of acute cardiogenic pulmonary edema
2. List the indications, contraindications, and risks associated with intubating a patient with acute cardiogenic pulmonary edema

**Case**: A 60-year-old man arrives to the Emergency Department by private vehicle with a chief complaint of shortness of breath that has progressively worsened over the past hour. He has a history of hypertension, diabetes, chronic obstructive pulmonary disease, and coronary artery disease. His vital signs include: afebrile, pulse 110, blood pressure 230/110, respiratory rate 28, pulse-ox 86% on room air. He is awake and speaks in short bursts of 3–5 words. He has a cough with intermittent pink thin frothy sputum. Lung exam reveals bilateral crackles and expiratory wheeze. Cardiac exam reveals a regular tachycardic rhythm without murmur. His skin is pink and mildly diaphoretic.

**Epidemiology**: Cardiogenic vs Non-cardiogenic (direct and indirect lung injury) pulmonary edema. Acute hospital mortality 10%–25% (especially if associated with myocardial infarction). One-year mortality >40%.

**Common causes of cardiogenic pulmonary edema**: acute congestive heart failure exacerbation, acute valve dysfunction, arrhythmia, hypertensive crisis, fluid overload following aggressive volume restriction, myocardial infarction, pericardial tamponade, ventricular septal rupture.

**Normal pulmonary interstitial fluid shift physiology:** There is a normal small net physiologic flow of fluid from the capillaries into the lung interstitial space mediated by hydrostatic and oncotic pressures and offset by efflux via pulmonary lymphatic channels. In the absence of lung injury, hydrostatic pressure is the primary driver of changes in the rate of fluid flux. Alveolar cell “tight junctions” normally prevent interstitial fluid from entering into the alveoli.

**Pathophysiology of cardiogenic pulmonary edema**: Increased hydrostatic pressure within the pulmonary capillaries leads to increased transvascular fluid filtration, primarily due to volume overload or impaired left ventricular function. Left atrial pressure increases result in interstitial space edema and when pulmonary capillary wedge pressure >25 mmHg, lymphatic and interstitial space capacity (+/− 500 mL) for fluid is overwhelmed and protein-poor fluid streams into the alveoli, impairing surfactant and destabilizing the alveolar units, resulting in hypoxia, VQ (ventilation/perfusion) mismatch, and cough productive of pink frothy thin sputum.

**Common symptoms and signs of pulmonary edema**: acute dyspnea, anxiety, orthopnea, pink frothy sputum, increased sympathetic tone manifested as tachycardia & hypertension, tachypnea, accessory muscle use, diaphoresis, lung exam with crackles and cardiac wheeze, peripheral cyanosis.

**Clinical studies that may aid diagnosis in the ED**: EKG (acute myocardial infarction, arrythmia), troponin, BNP (>1200 pg/mL favors cardiogenic pulmonary edema), BMP (acute kidney injury in the setting of fluid overload), portable CXR.

**Immediate ED stabilization/management (including risks and benefits of positive pressure ventilation and indication/contraindication for Endotracheal intubation (ETI)**: ABCs. Non-invasive positive pressure ventilation (NIPPV) vs ETI (work of breathing intolerable by patient, altered mental status). Code status may affect airway intervention. Supplemental oxygen. Positive pressure ventilation decreases preload and afterload, and splints open alveoli to improve VQ matching, oxygen diffusion, and carbon dioxide exchange. Nitrates (sublingual and intravenous). Loop diuretic and ACE inhibitor. Treat the underlying cause.

**Intubation adjuncts/techniques in acute pulmonary edema patients (instructor may choose to demonstrate)**: Maximize situation for success. Pre-oxygenate with NIPPV with patient in seated/Semi-Fowler’s position and FiO2 of 100%. Nasal cannula O2 for passive diffusion during intubation. Visualization challenges with direct vs videolaryngoscopy. Large bore hard Yankauer suction and gum bougie discuss/demonstrate. SALAD-like technique where the suction is parked in the hypopharynx to provide continuous suction. Seldinger technique whereby the large bore straight suction is advanced past the vocal cords and a bougie is passed through the suction catheter, creating a guide for an endotracheal tube.

**Source**: Besecker B and Crouser E. Pulmonary edema. In: Vincent J, Abraham E, Moore F, Kochanek P, Fink M, eds. *Textbook of Critical Care*. 7th ed. Philadelphia, PA: Elsevier Inc.; 2017:38–41.
